# Light-Programmable g-C_3_N_4_ Microrobots with Negative Photogravitaxis for Photocatalytic Antibiotic Degradation

**DOI:** 10.34133/research.0565

**Published:** 2025-01-28

**Authors:** Yunhuan Yuan, Xianghua Wu, Bindu Kalleshappa, Martin Pumera

**Affiliations:** ^1^ Future Energy and Innovation Laboratory, Central European Institute of Technology, Brno University of Technology, Brno 61200, Czech Republic.; ^2^ Department of Medical Research, China Medical University Hospital, China Medical University, Taichung TW-40402, Taiwan.; ^3^ Advanced Nanorobots & Multiscale Robotics Laboratory, Faculty of Electrical Engineering and Computer Science, VSB – Technical University of Ostrava, Ostrava 70800, Czech Republic.

## Abstract

Microrobots enhance contact with pollutants through their movement and flow-induced mixing, substantially improving wastewater treatment efficiency beyond traditional diffusion-limited methods. g-C_3_N_4_ is an affordable and environmentally friendly photocatalyst that has been extensively researched in various fields such as biomedicine and environmental remediation. However, compared to other photocatalytic materials like TiO_2_ and ZnO, which are widely used in the fabrication of micro- and nanorobots, research on g-C_3_N_4_ for these applications is still in its early stages. This work presents microrobots entirely based on g-C_3_N_4_ microtubes, which can initiate autonomous movement when exposed to ultraviolet and visible light. We observed distinct motion behaviors of the microrobots under light irradiation of different wavelengths. Specifically, under ultraviolet light, the microrobots exhibit negative photogravitaxis, while under visible light, they demonstrate a combination of 3-dimensional motion and 2-dimensional motion. Therefore, the wavelength of the light can be used for programming the motion style of the microrobots and subsequently their application. We show that the microrobots can effectively degrade the antibiotic tetracycline, displaying their potential for antibiotic removal. This exploration of autonomous motion behaviors under different wavelength conditions helps to expand research on g-C_3_N_4_-based microrobots and their potential for environmental remediation.

## Introduction

Owing to microrobots’ responsiveness, minimal invasiveness, autonomous movement capabilities, and programmable functions, they are revolutionizing various fields of application such as biomedicine, sensing, and environmental remediation [1–5]. Among them, light-driven microrobots show great promise in photocatalytic degradation of pollutants, as light induction enhances their movement and activates their inherent catalytic properties [5–7]. Most importantly, microrobots can directly utilize sunlight for environmental remediation, leading them to a highly attractive self-sustainable technology for the future [6]. The most extensively studied photocatalytic systems for microrobots include titanium dioxide (TiO_2_), zinc oxide (ZnO), iron oxide (Fe_2_O_3_), and tungsten trioxide (WO_3_), which are often used to form Janus particles with metal layers (platinum and gold) [8–11]. The requirement of ultraviolet (UV) light to initiate motion limits their use with visible (Vis) light. Thus, researchers have also fabricated systems that can be activated by Vis light, such as copper(I) oxide@cadmium selenide (Cu_2_O@CdSe), silver phosphate (Ag_3_PO_4_), and bismuth (Bi)-based materials [12–14]. Despite these advancements, challenges remain, such as high costs and the significant use of metals in these systems.

Due to its high surface area, Vis-light absorption, tunable bandgap, stability, low cost, and metal-free structure, graphitic carbon nitride (g-C_3_N_4_) has become an excellent photocatalytic material with immense potential [15,16]. However, compared to extensively used materials like TiO_2_ and ZnO in the preparation of micro- and nanorobots, there are relatively few reports on the effective utilization of g-C_3_N_4_ for the preparation of such devices [11,17]. Our group has developed a tubular g-C_3_N_4_ micromotor that can decompose hydrogen peroxide (H_2_O_2_) into oxygen (O_2_) bubbles under light, propelling itself and detecting metal ions in solution [18]. Another group created a Pt-*g*-C_3_N_4_ phototactic micromotor, whose movement relies on a self-diffusiophoretic mechanism and surface modifications [19]. Additionally, g-C_3_N_4_@carbon microsphere micromotors, driven by Vis-light-induced bubble propulsion, have been demonstrated for efficient removal of organic pollutants [20]. Currently, most g-C_3_N_4_-based microrobots are powered by bubble propulsion, but those that rely on diffusiophoretic or self-electrophoresis mechanisms are limited to 2-dimensional (2D) motion, with research into 3-dimensional motion (3D) motion being scarce [17]. This limitation to 2D motion is not conducive to the motion-induced enhancement of fluid mixing, affecting pollutant removal in water [7]. Consequently, there is an urgent need to develop g-C_3_N_4_-based light-driven micro- and nanorobots that not only explore various motion modes but also enhance efficiency in light utilization to broaden their application scope.

**Fig. 1.**
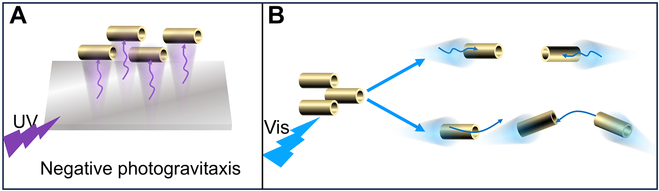
g-C_3_N_4_ microrobots exhibit (A) negative photogravitaxis movement under ultraviolet (UV) light and (B) distinct motion behaviors under visible (Vis) light.

Herein, we prepared g-C_3_N_4_-based microrobots that can catalyze the decomposition of H_2_O_2_ under UV or Vis light to create a chemical gradient, effectively powering their autonomous movement. These microrobots are prepared using inexpensive and easily accessible materials and simple equipment, making them ideal for mass production. We will show that the microrobots exhibit different motion behaviors under different wavelengths (Fig. 1). Under UV light, the microrobots demonstrate negative photogravitaxis (Fig. 1A). However, under Vis light, some microrobots perform pseudo-2D motion, while others achieve 3D motion (Fig. 1B). Therefore, using different wavelengths, we can program and switch the motion style of the microrobots. We will show that these microrobots can effectively break down the broad-spectrum antibiotic tetracycline (TC), markedly contributing to environmental remediation. Our research not only deepens the understanding of the movement patterns of g-C_3_N_4_ microrobots but also leads to their application in environmental remediation.

## Results and Discussion

**Fig. 2.**
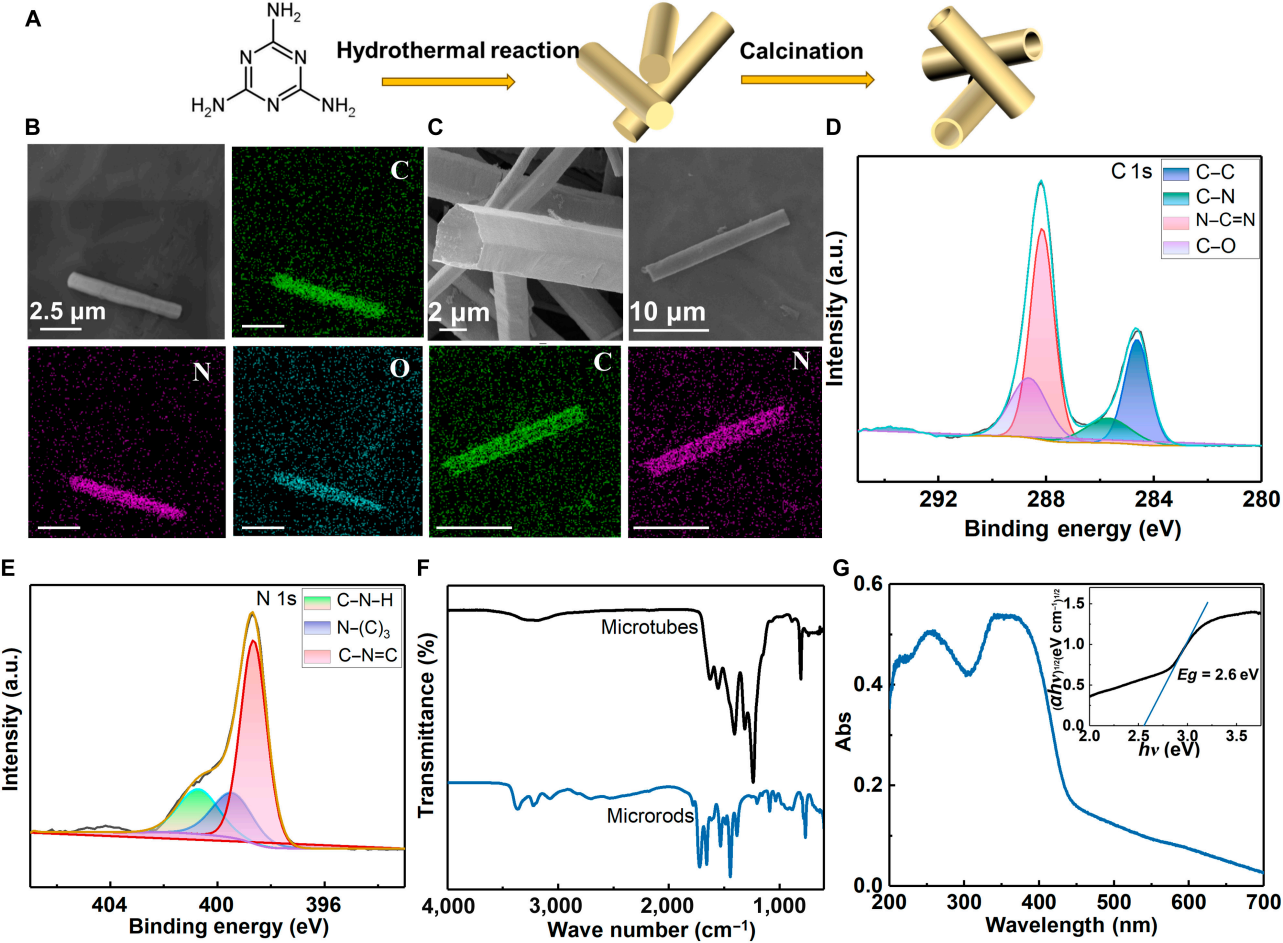
Synthesis and characterization of g-C_3_N_4_ microtubes. (A) The preparation scheme of g-C_3_N_4_ microtubes. (B) Scanning electron microscopy (SEM) and energy-dispersive x-ray (EDX) mapping images of the microrods (microrods are precursor products in the synthesis process of g-C_3_N_4_ microtubes, as shown in (A)). (C) SEM and EDX mapping images of the g-C_3_N_4_ microtubes (g-C_3_N_4_ microtubes are microrobots). (D) C 1s x-ray photoelectron spectroscopy (XPS) spectrum and (E) N 1s XPS spectrum of g-C_3_N_4_ microtubes. (F) Fourier transform infrared spectroscopy (FTIR) spectra of g-C_3_N_4_ microtubes. (G) The UV–Vis absorption spectra (inset: determining the bandgap energy (*Eg*) using a Tauc plot involves extrapolating the linear portion of the plot to the *x* axis). Abs, absorbance.

To obtain the final microtubes, precursor microrods were first synthesized from melamine via a hydrothermal process (Fig. 2A). During the hydrothermal process, part of the melamine is hydrolyzed into cyanuric acid, which then combines with melamine to self-assemble and connect through multiple hydrogen bonds to form microrods [21]. The resulting white powder primarily consists of microrods with diameters ranging from about 0.5 to 3.5 μm (Fig. S1), and the energy-dispersive x-ray (EDX) mapping images reveal that the microrods are mainly composed of the elements carbon (C), nitrogen (N), and oxygen (O) (Fig. 2B and Fig. S2). The x-ray diffraction (XRD) diffractograms in Fig. S3 illustrate that the primary peaks of the microrods are located at 10.8° (100) and 27.9° (002). According to previous reports, the peak at (100) is associated with in-planar stacking, while the peak at (002) corresponds to interlayer stacking [22]. The result indicates that this microrod intermediate has a layered structure similar to that of typical g-C_3_N_4_ [23]. After polycondensation under a nitrogen (N_2_) atmosphere, microrods transform into a yellow powder g-C_3_N_4_ microtubes, which are primarily composed of C and N. Figure 2C clearly illustrates that the external tubular morphology is well-preserved and also matches the size of the microrods, with diameters primarily ranging from about 0.5 to 3.5 μm and lengths mainly around 5 to 25 μm (Fig. S1). During the calcination, the inner core of the microrods gradually disappears, leading to the formation of hollow structures. This is due to the formation of defects at the center of the supramolecular intermediate during the early stages of the hydrothermal reaction [24]. Compared to traditional bulk graphitic carbon nitride (JCPDS card: 87-1526), the (002) peak of the microtubes is broader and weaker due to the reduced number of layers, which has a larger interlayer spacing. Another significant (100) peak of bulk graphitic carbon nitride is invisible in microtubes because of the smaller planes of the layers [23,24].

X-ray photoelectron spectroscopy (XPS) was employed to measure and analyze the chemical composition of the sample. Figure S4 illustrates that the primary composition of the tube is made of carbon and nitrogen. The C 1s spectrum reveals that the 4 main peaks located at 284.6, 285.7, 288.2, and 288.7 eV can be assigned to graphitic carbon (C–C), the C–N bond, the sp^2^-hybridized carbon in the N-containing aromatic ring (N–C=N), and the C–O bond, respectively (Fig. 2D). For the N 1s spectrum, the broad peak ranging from 396 to 403 eV can be deconvoluted into peaks at 398.7, 399.5, and 400.8 eV, corresponding respectively to sp^2^-hybridized nitrogen in triazine rings (C–N=C), tertiary nitrogen N–(C)_3_ groups, and terminal amino groups (C–N–H). Additionally, a small peak at 404.2 eV is associated with the localization of positive charge in heterocycles (Fig. 2E) [21,25,26]. Fourier transform infrared spectroscopy (FTIR) was used to identify the functional groups of the intermediate rods and the final tubes. Figure 2F demonstrates that the peaks between 3,000 and 3,400 cm^−1^ result from the stretching vibrations of N–H bonds in –NH_2_ and O–H bonds of residual hydroxyl groups or adsorbed H_2_O. The peaks in the range of 1,200 to 1,600 cm^−1^ can be attributed to the vibrations of aromatic CN heterocycles, while the peak around 810 cm^−1^ originates from the characteristic bending vibration of *s*-triazine subunits. Compared to the intermediate rods, the tubes exhibit very weak peaks between 3,000 and 3,400 cm^−1^ after undergoing thermal polymerization, indicating intensified condensation during the synthesis process [27,28]. Additionally, the intermediate rods exhibit extra peaks at 1,725 and 1,100 cm^−1^, corresponding to C=O and C–O stretching vibrations, respectively [29]. This indicates that the intermediate microrods contain more oxygen-containing functional groups compared to the microtubes. Additionally, the XPS spectra reveal that the microrods contain more oxygen than the microtubes, with oxygen atomic percentages of 13.9% and 2.7%, respectively (Fig. S4). UV–Vis spectroscopy was employed to evaluate the light absorption properties and bandgap of g-C_3_N_4_. Figure 2G shows significant absorption between 337 and 373 nm, with the photoabsorption edge extending up to 448 nm. The bandgap is obtained using the Tauc method, with the formula (*αhν*)^1/*n*^ = *A*(*hν* − *Eg*), where *α* is the absorption coefficient, *h* is Planck’s constant, *ν* is the light frequency, *A* is a constant, *Eg* is the energy bandgap, and *n* corresponds to the electronic transition in semiconductors. For direct and indirect transitions, *n* is equal to 1/2 and 2, respectively [30–32]. Since g-C_3_N_4_ is generally considered an indirect semiconductor, *n* is taken as 2 [32,33]. From the Tauc plot (inset of Fig. 2G), the bandgap (*Eg*) of g-C_3_N_4_ microtubes is determined to be 2.6 eV. This is obtained by extrapolating the linear portion to the *x* axis, where the intersection point with the *x* axis provides an estimate of the bandgap [30–32]. This reduction in bandgap could be due to multiple scattering of light within the microtubes. A narrower bandgap enables easier excitation of the microtubes by longer wavelengths of sunlight, increasing the generation of photogenerated electrons and holes and thereby improving the utilization efficiency of sunlight [28].

**Fig. 3.**
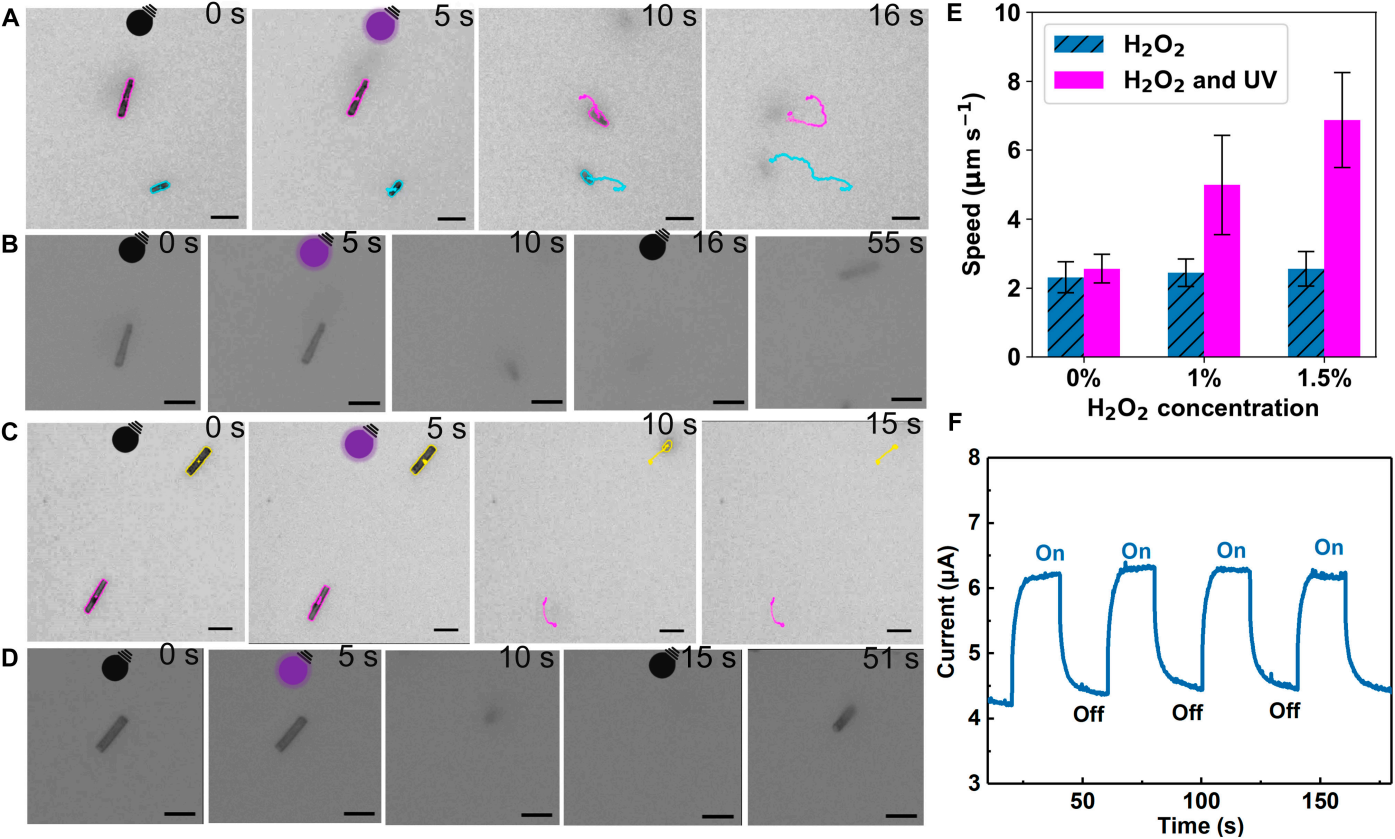
Motion behavior of the microrobots under UV light. (A) Light-dependent motion trajectories of microrobots with 1 wt% H_2_O_2_ (dark: 0 to 5 s; light: 5 to 16 s). (B) Micrographs depicting typical movements of microrobots under alternating dark and UV-light conditions with 1 wt% H_2_O_2_ (dark: 0 to 5 and 16 to 55 s; light: 5 to 16 s). (C) Light-dependent motion trajectories of microrobots with 1.5 wt% H_2_O_2_ (dark: 0 to 5 s; light: 5 to 15 s). (D) Micrographs showing typical movements of microrobots under alternating dark and light conditions with 1.5 wt% H_2_O_2_ (dark: 0 to 5 and 15 to 51 s; light: 5 to 15 s). (E) Speed of microrobots as a function of H_2_O_2_ concentration and UV irradiation. (F) Photocurrent density of the microrobots under light on and off modes. (All scales are 10 μm.)

Further, we explored the motion behaviors of microrobots (g-C_3_N_4_ microtubes) in water using external 365-nm UV-light sources and 400-nm Vis-light sources, with representative videos of their propulsion shown in Videos S1 to S6. Figure 3 illustrates the motion behaviors of microrobots in different concentrations of H_2_O_2_ under UV light. In 1 wt% H_2_O_2_, the microrobots moved rapidly under UV irradiation, quickly becoming unfocused and completely disappearing around 11 s, suggesting upward movement. When the UV light was turned off, the microrobots began to refocus and settle at the bottom of the microscope slide, remaining in focus within 35 to 40 s (Fig. 3A and B and Video S1). The motion behaviors with 1.5 wt% H_2_O_2_ under UV off–on conditions were similar (Fig. 3C and D and Video S2). This type of motion is classified as negative photogravitaxis, where the light-driven force and buoyancy overcome gravity, enabling the microrobots to move upward when illuminated vertically from the bottom of the substrate [34,35]. This negative photogravitaxis movement in microrobots, which is not limited to planar motion, enhances fluid mixing, increasing the interaction between pollutants and the microrobot surfaces, and ultimately improves the efficiency of pollutant removal from water [7].

Figure 3E also presents statistics on the speed of microrobots on the *XY* axis under various conditions, with each dataset involving over 30 particles. In 0 wt% H_2_O_2_, the motion speed of the microrobots changed very little under dark and UV-light conditions (Video S3). However, under UV light with 1 and 1.5 wt% H_2_O_2_, the average speeds were 2.1 and 2.7 times that of the dark conditions, reaching 5 and 6.87 μm s^−1^, respectively. The photocatalytic material catalyzes the decomposition of H_2_O_2_ under light exposure, producing chemical species, such as •O_2_^−^, •OH, and ^1^O_2_. To identify the types of chemical species produced, we conducted electron paramagnetic resonance (EPR) experiments. 5-*tert*-Butoxycarbonyl-5-methyl-1-pyrroline-*N*-oxide (BMPO) was used as the trapping reagent for •O_2_^−^ and •OH, while 2,2,6,6-tetramethyl-4-piperidinol (TMP) was employed to trap ^1^O_2_. Under illumination, the EPR signal of the BMPO adducts was substantially enhanced (Fig. S5a). The illuminated spectra were subsequently simulated using EasySpin, an open-source MATLAB toolbox for simulating and fitting EPR spectra [36]. Figure S5b presents the spin-trapped EPR spectra, the simulation spectra, and the fitted spectra of each radical adduct under illumination. The results indicate that in the presence of BMPO, 2 spin adducts (BMPO-superoxide and BMPO-hydroxyl) were formed, each consisting of 2 stereoisomers. The above results demonstrate that microrobots indeed produce •O_2_^−^ and •OH under illumination [37,38]. TMP is an EPR-silent species that reacts with ^1^O_2_ to form the EPR-active species 4-hydroxy-2,2,6,6-tetramethylpiperidine 1-oxyl. From this trapping experiment, we confirmed the production of more ^1^O_2_ under illumination by the microrobots (Fig. S6). The uneven distribution of these species induces diffusiophoretic propulsion of the particles, resulting in a rapid increase in the speed of the microrobots [18,34,39]. The transient photoelectrochemical response demonstrates the semiconductor’s capability for photoinduced charge transfer and separation [40]. Thus, the photocurrent of microrobots was measured using a 3-electrode system at an electrochemical workstation. As depicted in Fig. 3F, the photocurrent remained stable and exhibited excellent repeatability. When the light was switched off, the photocurrent quickly decreased; however, it immediately increased upon re-exposure to light, demonstrating the photoresponse characteristics of the microrobots and fast charge transport, which enhances the material’s photocatalytic performance.

**Fig. 4.**
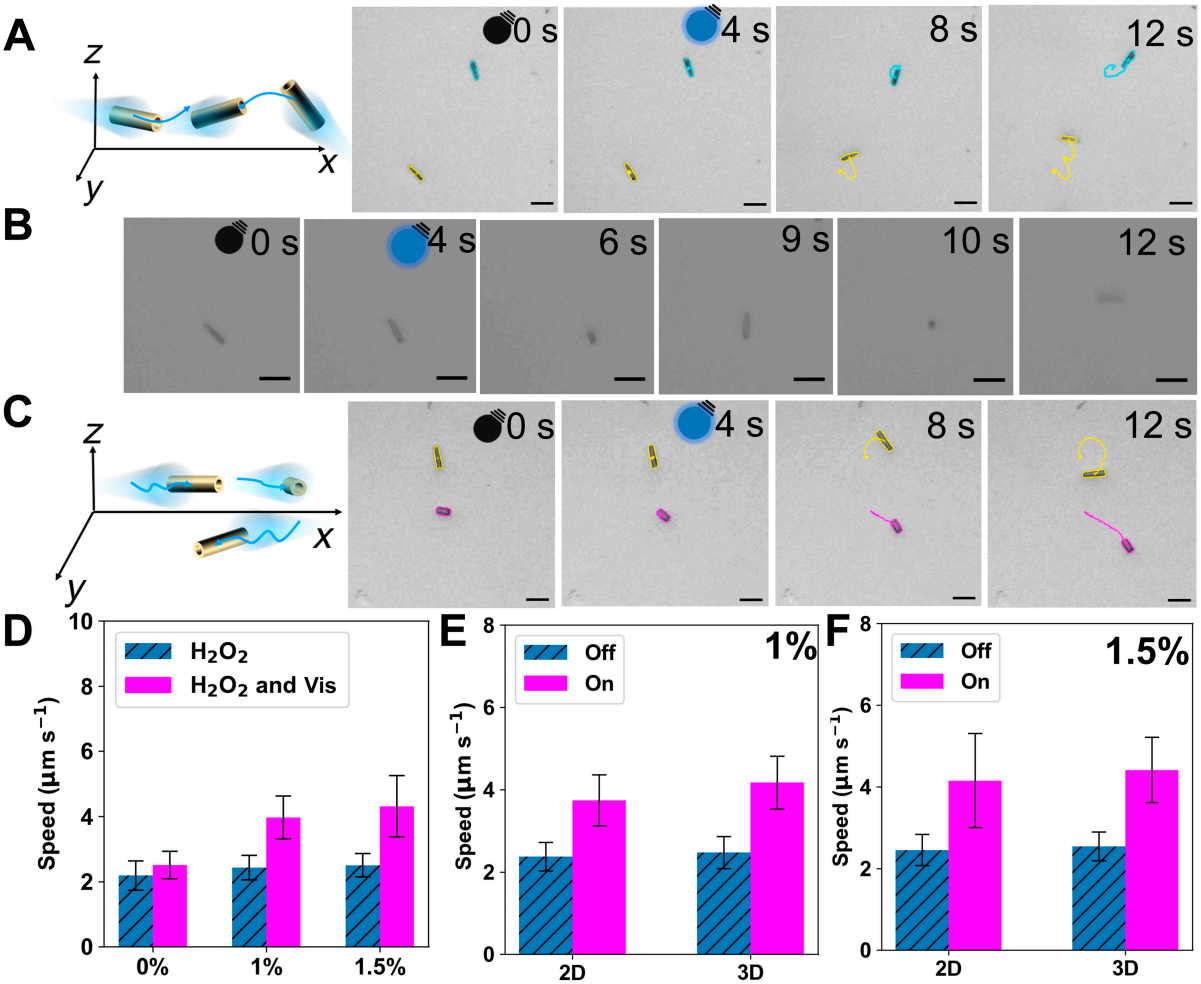
Motion behavior of the microrobots under Vis irradiation. (A) Light-dependent 3-dimensional (3D) motion trajectories of microrobots (dark: 0 to 4 s; light: 4 to 12 s). (B) Micrographs depicting typical 3D motion of microrobots under dark and Vis conditions (dark: 0 to 4 s; light: 4 to 12 s). (C) Light-dependent 2-dimensional (2D) motion trajectories of microrobots (dark: 0 to 4 s; light: 4 to 12 s). (D) Microrobots’ speed as a function of H_2_O_2_ concentration and Vis irradiation. (E) Speed statistics of microrobots performing 2D and 3D motions with 1 wt% H_2_O_2_. (F) Speed statistics of microrobots performing 2D and 3D motions with 1.5 wt% H_2_O_2_. (All scales are 10 μm.)

A 400-nm light source was employed to explore the impact of Vis light on the movement of microrobots. As depicted in Fig. 4, the microrobots exhibit different modes of motion under Vis light, as detailed in Videos S4 to S6. When exposed to light, the microrobots moved rapidly, with some moving in 3 dimensions (Fig. 4A and B and Video S4) and others moving in 2 dimensions (Fig. 4C and Video S5), and those in 3 dimensions did not completely disappear. Additionally, the movement speeds of more than 50 microrobots in both 1 and 1.5 wt% H_2_O_2_ were statistically analyzed. According to Fig. 4D, the average speeds of the microrobots under light was around 1.7 times that of the dark conditions. It is noteworthy that according to standards in this field, the speed of microrobots performing 3D motion is calculated based only on their projections on the *xy* plane [35]. Additionally, the percentages of particles moving in 2 and 3 dimensions were also statistically analyzed. Specifically, in 1 wt% H_2_O_2_, particles moving in 2 dimensions accounted for 44%, while those moving in 3 dimensions accounted for 56% (Fig. 4E). Moreover, in 1.5 wt% H_2_O_2_, the particles moving in 2 and 3 dimensions accounted for 36% and 64%, respectively (Fig. 4F).

**Fig. 5.**
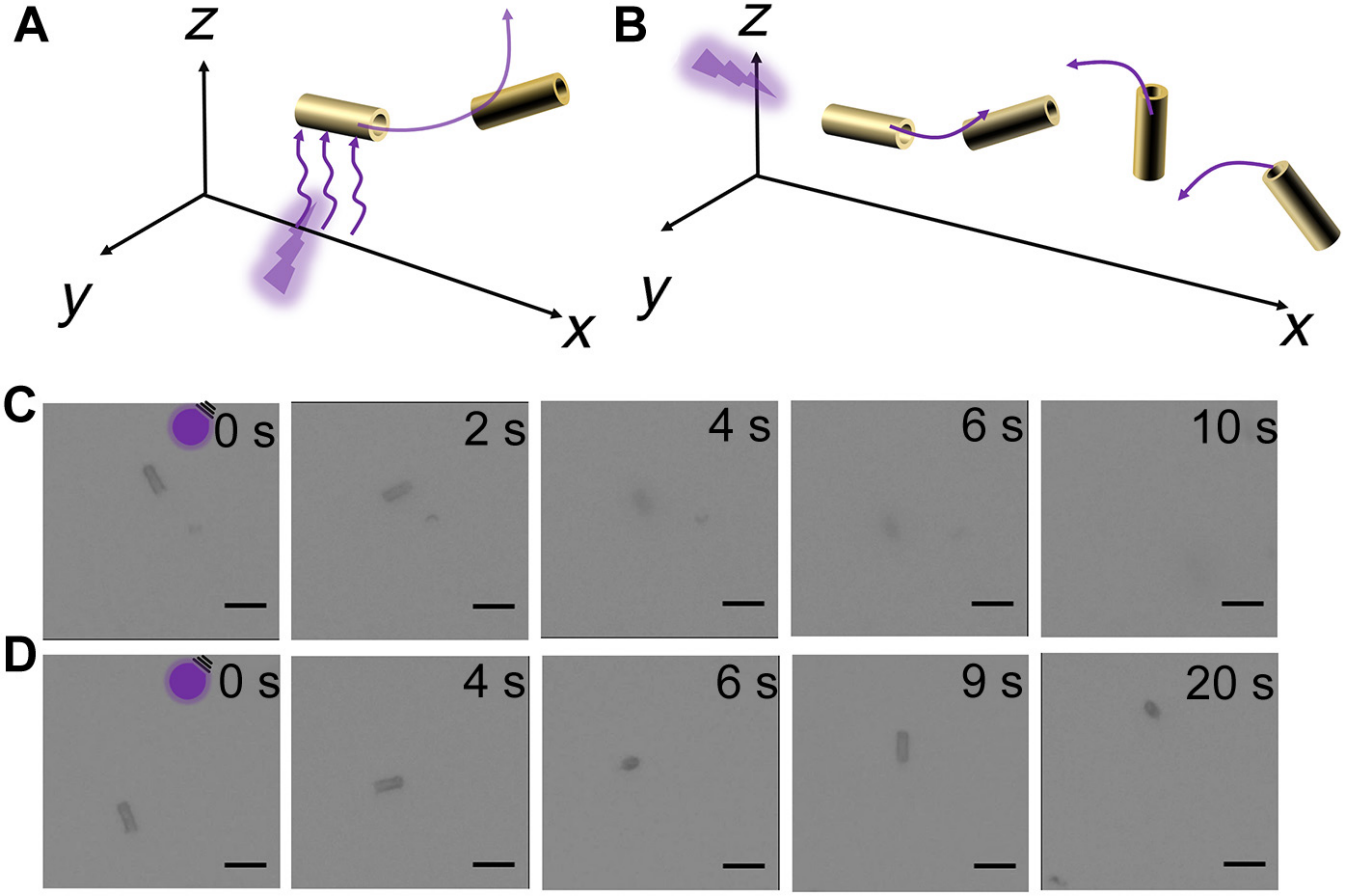
Analysis of the motion mechanisms of microrobots. (A) Schematic illustration of microrobot motion when illuminated from the bottom. (B) Schematic illustration of microrobot motion when illuminated from the left side. (C) Micrographs showing microrobot motion when vertically illuminated with UV light from the bottom of the substrate. (D) Micrographs showing microrobot motion when illuminated with UV light from the left side. (All scales are 10 μm.)

Because of the different motion behaviors of microrobots under UV and Vis light, a more detailed discussion of the propulsion mechanism should be explored. It is well-known that g-C_3_N_4_, as a semiconductor, excites electrons from the valence band (VB) to the conduction band (CB) through light absorption, leaving holes in the VB and effectively separating photogenerated carriers. Figure 3F displays the charge transfer and separation capabilities of these microrobots. These electrons and holes that accumulated on the surface of the microrobots can rapidly react with H_2_O_2_ to form a chemical gradient, resulting in diffusiophoretic propulsion [34]. Due to the structural morphology and homogeneity of the material, along with the direction of light irradiation, chemical gradients form asymmetrically around the microrobots, potentially triggering their propulsion [34,41]. Figure 5 illustrates the different motion behaviors of the microrobots under the UV (365-nm) light irradiation from different angles (bottom and left side). When UV light irradiates from the bottom, a higher concentration of electrons and holes at the base of the microrobot rapidly reacts with H_2_O_2_ (1.5 wt%), creating a chemical gradient. This gradient, combined with buoyancy, completely overcomes gravity, thereby initiating the upward movement of the microrobot (Fig. 5A and C and Video S7). However, when the UV light comes from the left side, electrons and holes accumulate unevenly only on the surface, without forming a high concentration at the base, thus failing to completely counteract gravity for upward motion (Fig. 5B and D and Video S8). Under Vis-light irradiation, the energy is lower than that of UV light, which also proves insufficient to overcome gravity and achieve upward movement.

Subsequently, we investigate the ability of microrobots to enhance the degradation of TC. TCs are widely used in human medicine and veterinary practices because of their broad-spectrum antimicrobial properties. They can effectively treat a variety of bacterial infections, including skin infections, urinary tract infections, and pneumonia. However, their widespread use causes environmental challenges, especially when they can contaminate aquatic ecosystems. Since TCs do not easily degrade in natural environments, they can accumulate in water bodies, leading to serious adverse effects on aquatic life. These include increasing bacterial resistance, inhibiting algal growth, and impairing fish reproduction and growth. As time increases, such ecological imbalances will threaten the health and stability of entire aquatic food chains. Hence, it is crucial to develop efficient, eco-friendly, and cost-effective technologies to remove these antibiotics from aquatic environments, thereby protecting water quality and the health of aquatic ecosystems [42].

**Fig. 6.**
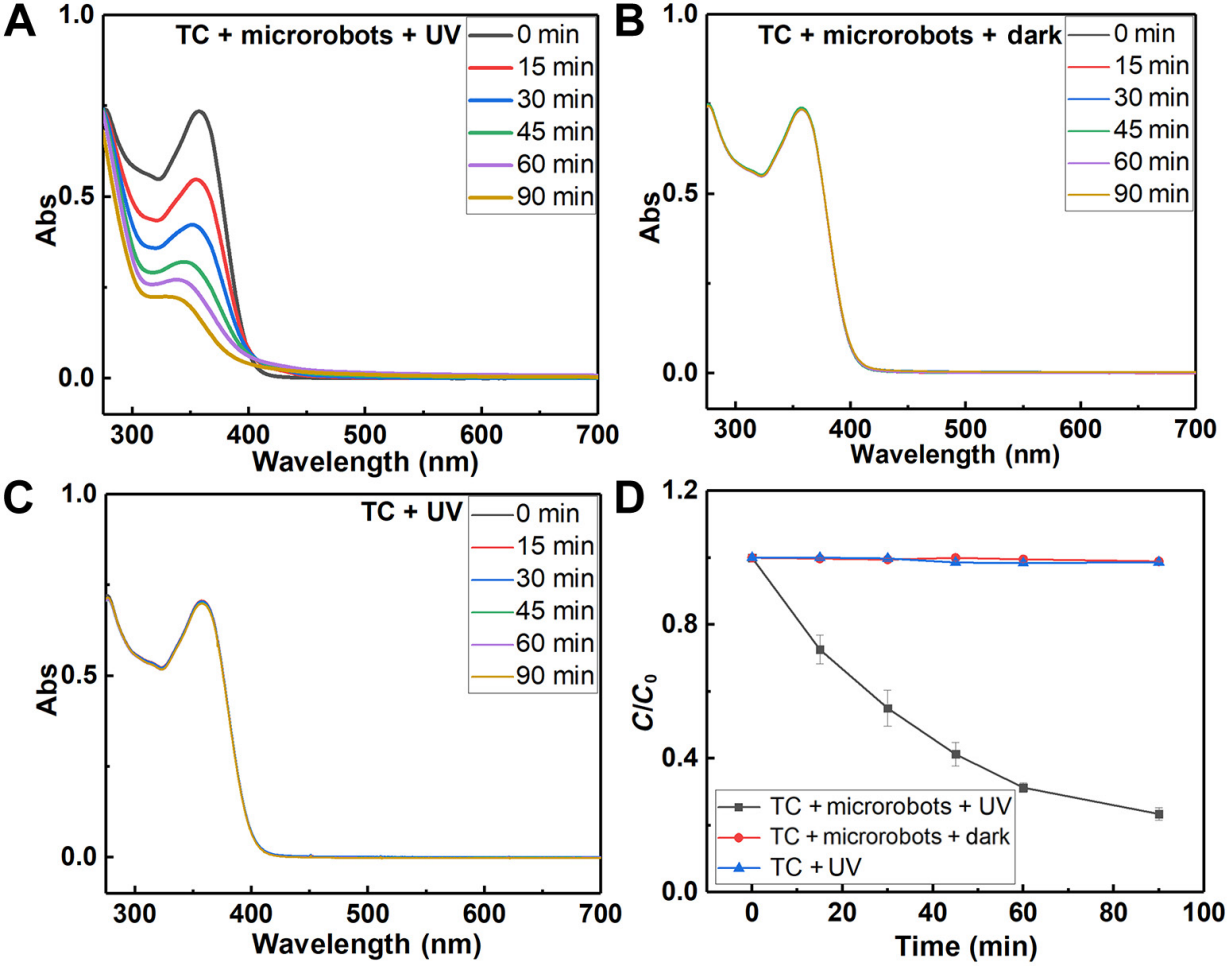
Degradation of tetracycline under different conditions. Time-dependent absorption spectra of tetracycline solution (A) under UV-light irradiation in the presence of microrobots, (B) in the dark with microrobots, and (C) under UV-light irradiation without microrobots. (D) Degradation dynamics curves of tetracycline solution under various conditions (*N* = 3). TC, tetracycline.

We explore the photocatalytic properties of microrobots for degrading TC. After adsorption–desorption equilibrium was reached between the microrobots and TC, stirring was stopped, and UV light was activated. Figure 6A presents the absorption spectra of TC solutions over time under UV light, with microrobots serving as the photocatalyst. The data reveal a significant reduction in TC absorbance with increased irradiation time. In contrast, control groups, including TC in the presence of microrobots under dark conditions (Fig. 6B) and TC exposed to UV light in the absence of microrobots (Fig. 6C), showed minimal absorbance reduction. Figure 6D shows the degradation dynamics, highlighting a significant reduction in TC concentration as the UV irradiation time increased, reaching a removal efficiency of 77% within 90 min. The control groups, however, exhibited extremely low removal efficiencies. These findings reveal the excellent removal capabilities of microrobots for TC. It is important to note that pure g-C_3_N_4_ typically lacks the efficiency needed to degrade organic pollutants effectively. This limitation has prompted many researchers to incorporate cocatalysts to enhance the performance of g-C_3_N_4_-based composite photocatalysts [43]. However, this strategy complicates the synthesis process, resulting in structurally complex and expensive photocatalysts. Such complexities are not ideal for large-scale industrial production. The g-C_3_N_4_ microtubes (microrobots) developed in this work do not require complex synthesis processes or toxic elements, making them cost-effective and suitable for large-scale production [43]. The results confirm that these affordable and readily available microrobots can effectively degrade TC, demonstrating significant potential for practical applications in aquatic environments. According to the existing literature, the final products of the photocatalytic degradation of TC using g-C_3_N_4_ materials have little harm to the environment and living organism [44–46]. Most of the products are ultimately converted into carbon dioxide, water, and other low-molecular-weight compounds [44–46]. Furthermore, to demonstrate that enhanced fluid motion indeed increases degradation efficiency, we conducted experiments with slow stirring to simulate enhanced fluid mixing. The results showed a slight improvement in degradation efficiency (79%) compared to static conditions (Fig. S7).

**Fig. 7.**
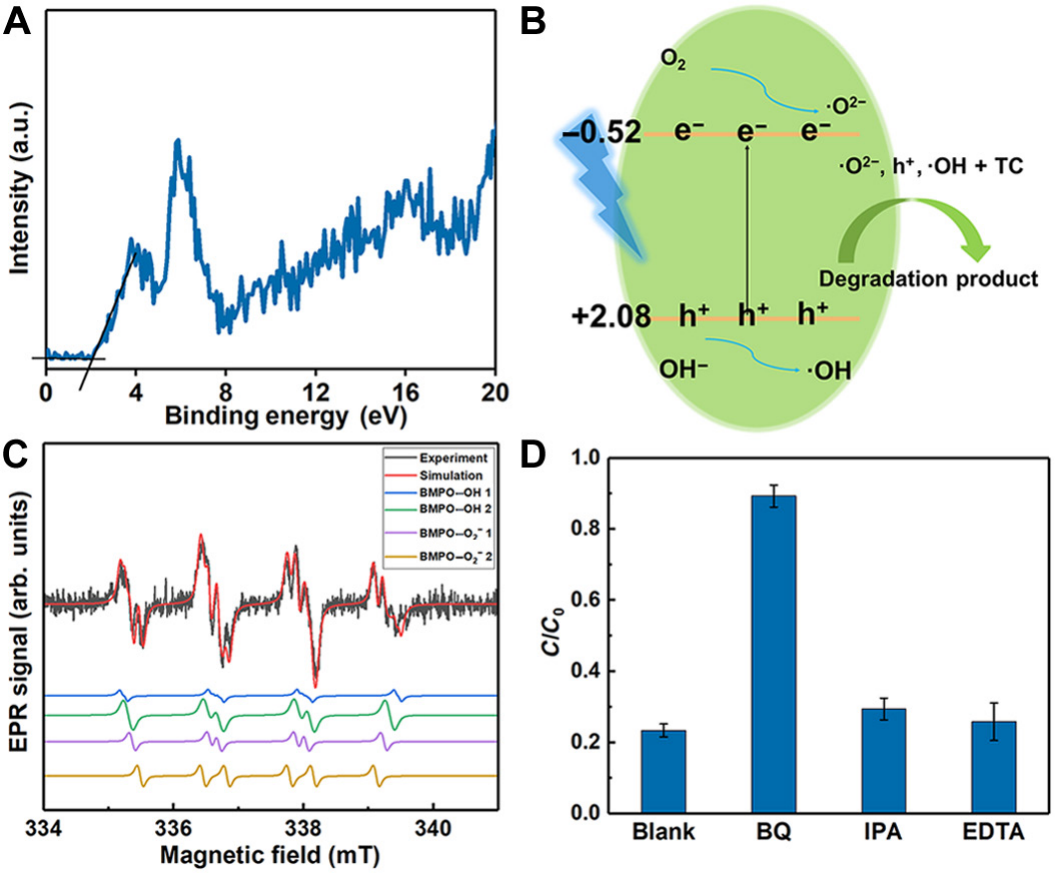
The mechanism of TC degradation by microrobots. (A) XPS valence band analysis. (B) Schematic illustration of the photodegradation mechanism of TC by microrobots. (C) Spin-trapped electron paramagnetic resonance (EPR) spectra, the simulation spectra, and the fitted spectra of each radical adduct under illumination. (D) Degradation efficiency of TC using microrobots in the presence of different scavengers. IPA, isopropanol.

The mechanism of g-C_3_N_4_ photocatalytic degradation of organic pollutants in water has been extensively studied and discussed [27,47,48]. Generally, the active species involved in this degradation process include superoxide radicals (•O_2_^−^), hydroxyl radicals (•OH), and photogenerated holes (h^+^) [27,47,48]. To clarify the mechanism, the potentials of the CB and VB were determined using XPS VB analysis and the bandgap energy (*Eg*) [49]. As shown in Fig. 7A, the VB is located at +2.08 eV. Based on previous results (Fig. 2G), the bandgap (*Eg*) of the microtubes was determined to be 2.6 eV; therefore, the CB is at −0.52 eV (*E*_VB_ = *E*_CB_ + *Eg*) [49]. In this study, both light and microrobots play critical roles in achieving high degradation efficiency. The proposed mechanism, depicted in Fig. 7B, begins with g-C_3_N_4_ absorbing light, which excites electrons from the VB to the CB, leaving holes in the VB. This process effectively separates the photogenerated carriers. The excited electrons then react with O_2_ to form •O^2−^, while the holes react with water to produce •OH [40,47,49]. It is important to note that the CB of microrobots, at −0.52 eV, is lower than the redox potential of O_2_/O^2−^ at −0.33 eV versus the reversible hydrogen electrode, allowing the reaction O_2_ + e^−^ → •O^2−^ to proceed. Moreover, the VB of +2.08 eV is more positive than that of •OH/OH^−^ (+1.99 eV), suggesting that the holes on the surface of microrobots may react with OH^−^ in H_2_O to generate •OH [40,49,50].

To confirm the presence of the reactive species •O_2_^−^ and •OH, we conducted EPR experiments. BMPO was used as the trapping reagent for •O_2_^−^ and •OH. As shown in Fig. S8, no significant signals were observed in the dark. However, upon illumination of the microrobots, significant signals appeared in the spectra, which were then simulated using EasySpin [36]. Figure 7C displays the spin-trapped EPR spectra, the simulation spectra, and the fitted spectra of each radical adduct. The results demonstrate that in the presence of BMPO, 2 spin adducts (BMPO-superoxide and BMPO-hydroxyl) were formed, with each adduct comprising 2 stereoisomers (Fig. 7C). These findings indicate that the primary components generated are •O_2_^−^ and •OH [37,38]. The above results demonstrate that microrobots indeed produce •O_2_^−^ and •OH under illumination.

Radical scavenging experiments were subsequently performed to further investigate the role of active species in the degradation of TC. Benzoquinone (BQ), isopropanol (IPA), and ethylenediaminetetraacetic acid (EDTA) were employed as scavengers for •O^2−^, •OH, and h^+^, respectively [40,51]. The degradation efficiency decreased to 11% upon the addition of BQ. In contrast, when IPA and EDTA were added individually, the degradation efficiencies remained relatively high at 70% and 76%, respectively, only slightly lower than the efficiency observed in the absence of scavengers (77%). These findings suggest that •O^2−^ is the primary active species responsible for TC degradation. In summary, microrobots facilitate pollutant degradation through redox reactions involving reactive species (O^2−^, h^+^, and •OH), with O^2−^ playing a major role in the process.

## Conclusion

This work demonstrates that g-C_3_N_4_ microrobots can change their motion behavior by altering the wavelength of light used. Under UV light, the microrobots demonstrate strong negative photogravitaxis. Conversely, under Vis light, they exhibit diverse motion behaviors, with some microrobots exhibiting 3D movement and others showing pseudo-2D movement. Additionally, these microrobots without toxic elements can effectively degrade TC, exhibiting their potential in antibiotic degradation. Overall, this research not only deepens our knowledge of the motion behaviors of g-C_3_N_4_ materials but also expands the applications of these microrobots in the area of environmental remediation.

## Materials and Methods

### Fabrication of g-C_3_N_4_ microtubes

To synthesize g-C_3_N_4_ microtubes (microrobots), 1 g of melamine was mixed into 100 ml of deionized water, followed by the addition of 0.4 g of NaOH. The solution was then heated to 70 °C and stirred continuously for 20 min. Subsequently, the solution was placed into a Teflon-lined autoclave and subjected to heating at 180 °C for 14 h in an oven. Upon natural cooling, the supernatant was discarded, and the resulting precipitate was collected. The precipitate was repeatedly washed with ethanol and deionized water and then dried at 50 °C in an oven to acquire white powder microrods, which served as the precursor material for synthesizing microtubes. This powder was then placed into a covered crucible and transferred to a tube furnace. In a nitrogen atmosphere, the material was heated to 500 °C at a rate of 5 °C·min^−1^ for 90 min. The resulting yellow powder is g-C_3_N_4_ microtubes.

### Characterization

Surface images and elemental compositions were obtained using a MIRA3 XMU scanning electron microscope equipped with an EDX detector. XRD patterns were acquired with Rigaku SmartLab. XPS measurements were conducted with a KRATOS Axis Supra instrument. UV–Vis absorption spectra were collected by a V-730 UV–Vis spectrophotometer. Notably, the UV–Vis diffuse reflectance spectroscopy method was employed to analyze the prepared g-C_3_N_4_ microtube powder, evaluating its light absorption properties and bandgap. The absorption spectrum (Fig. 2G) was subsequently calculated by the Jasco software based on the corresponding reflectance measurements. Functional groups were analyzed using a Vertex V70 Fourier transform infrared spectrometer.

### Photocurrent response measurement

To obtain the dispersion, 2 mg of g-C_3_N_4_ microtubes powder was combined with a mixture of 0.5 ml of water, 0.5 ml of ethanol, and 10 μl of Nafion (5%) and then sonicated for 1 min to ensure uniform dispersion. The resulting solution was evenly drop-cast onto screen-printed carbon electrodes (SPCEs) and dried. The g-C_3_N_4_ microtubes@SPCE was placed in a 0.5 M Na_2_SO_4_ electrolyte solution. Photocurrent measurements were conducted using a potentiostatic method at a potential of 0.8 V, with photocurrent density–time curves recorded. A platinum wire electrode served as the counter electrode, and an Ag/AgCl electrode served as the reference electrode. UV light was directly irradiated onto the surface of the sample from a light source positioned 10 cm to the side of the sample, with on/off switching every 20 s.

### Microrobots’ motion behavior

Microrobot motion experiments were performed using a Nikon ECLIPSE Ts2R inverted microscope, which was equipped with a BASLER acA1920-155uc digital camera. Illumination was provided by UV light-emitting diodes with wavelengths of 365 and 400 nm (CoolLED pE-100), each operating at 1,600 mW/cm^2^. Specifically, a 10-μl aqueous suspension of g-C_3_N_4_ microrobots was mixed with 10 μl of hydrogen peroxide at varying concentrations, resulting in final concentrations of 1 and 1.5 wt%. The behavior of the microrobots under illuminated and nonilluminated conditions was recorded. Videos were captured at a frame rate of 25 fps. Subsequently, the motion trajectories and speeds of the microrobots were analyzed using the NIS-Elements Advanced Research software. Each dataset included statistics from more than 30 particles, obtained from multiple corresponding videos. No surfactants were used throughout the experiments.

### Photocatalytic degradation of TC

The catalytic performance of microrobots was examined by degrading TC solution in a 20-ml glass vial. Specifically, 2 ml (1 mg/ml) of microrobots was mixed with a 15-ml TC aqueous solution (2.5 mg/100 ml). The suspension was stirred in the dark for 40 min to achieve adsorption–desorption equilibrium before irradiation. The light source (365 nm, 8 W) was then activated. At a given time, 2 ml of the suspension was withdrawn and filtered using a polyvinylidene fluoride membrane to separate microrobots. The absorbance of the filtered solution was determined using a UV–Vis spectrophotometer, with the absorption peak at 357 nm used to calculate the degradation efficiency. Control experiments were conducted to accurately assess the photocatalytic contribution of the microrobots. These controls involved keeping TC and microrobots solutions in the dark and irradiating solutions containing only TC. The removal efficiency of TC was calculated using the following formula: removal efficiency = (*C*_0_ − *C*)/*C*_0_ × 100%, where *C*_0_ is the initial concentration of TC and *C* is the concentration at a given time during the degradation process. Moreover, radical scavenging experiments were performed by adding 1 mmol of BQ (a quencher of •O_2_^−^), 1 mmol of IPA (a quencher of •OH), and 1 mmol of EDTA (a quencher of h^+^) to investigate the role of active species in TC degradation. The concentration of TC in the solution was determined using a UV–Vis spectrophotometer.

### EPR experiments

The EPR experiments were conducted using a Magnettech X-band EPR spectrometer equipped with a cuvette for aqueous samples (referred to as a flat cell). The spectra were recorded using a microwave power of 3.9 mW and a modulation amplitude of 0.04 mT at 100 kHz. Data simulation and fitting were conducted using EasySpin, an open-source MATLAB toolbox for EPR spectral analysis.

## Data Availability

The data that support the findings of this study are available at the www.zenodo.org repository.

## Supplementary Materials

Supplementary 1 Figs. S1 to S9 Videos S1 to S9
