# Supplementary figures and images for: Light-Programmable g-C3N4 Microrobots with Negative Photogravitaxis for Photocatalytic Antibiotic Degradation
Source: Research (Wash D C). 2025 Jan 28;8:0565. doi: 10.34133/research.0565 (PMC11772662; doi:10.34133/research.0565)

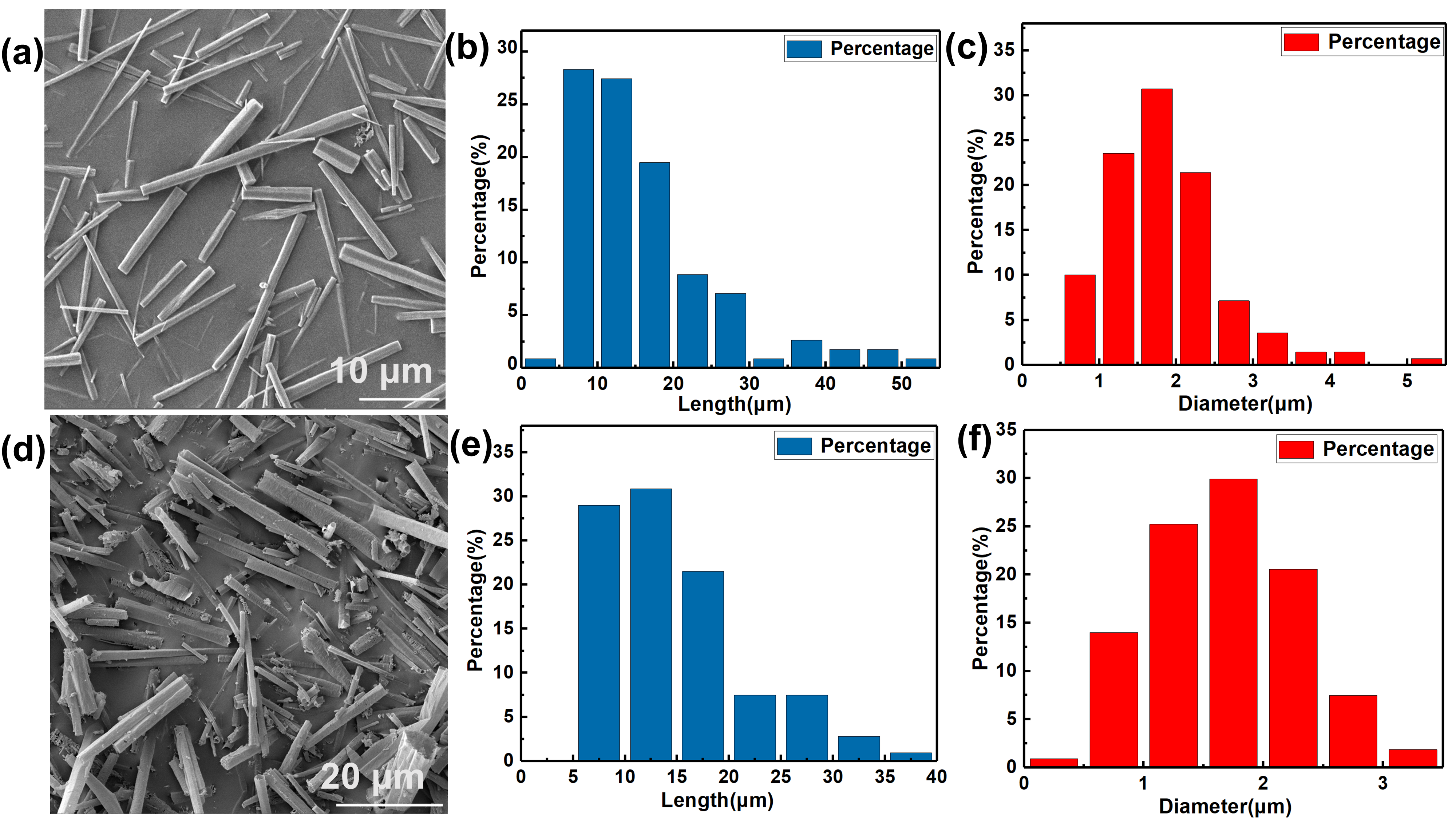

Supplement: Supplementary 1 — Figs. S1 to S9 Videos S1 to S9 [file research.0565.f1.zip › Figure S1.tif]

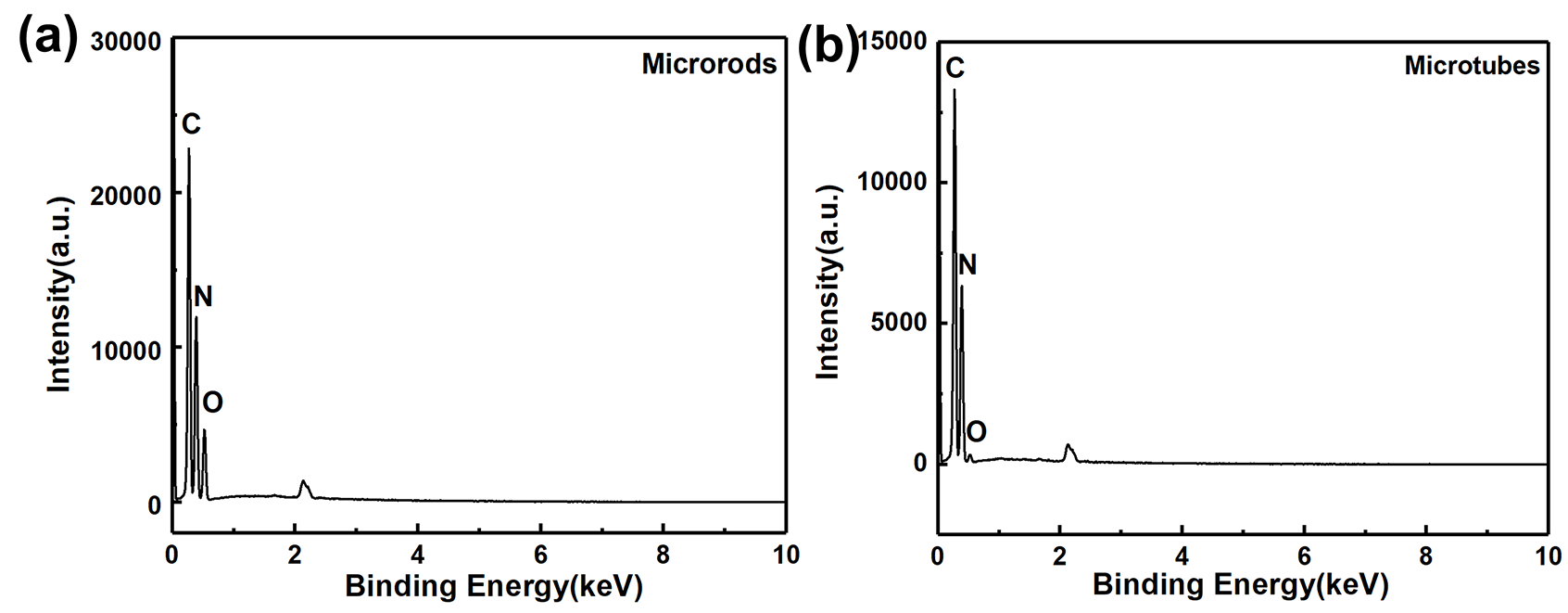

Supplement: Supplementary 1 — Figs. S1 to S9 Videos S1 to S9 [file research.0565.f1.zip › Figure S2.tif]

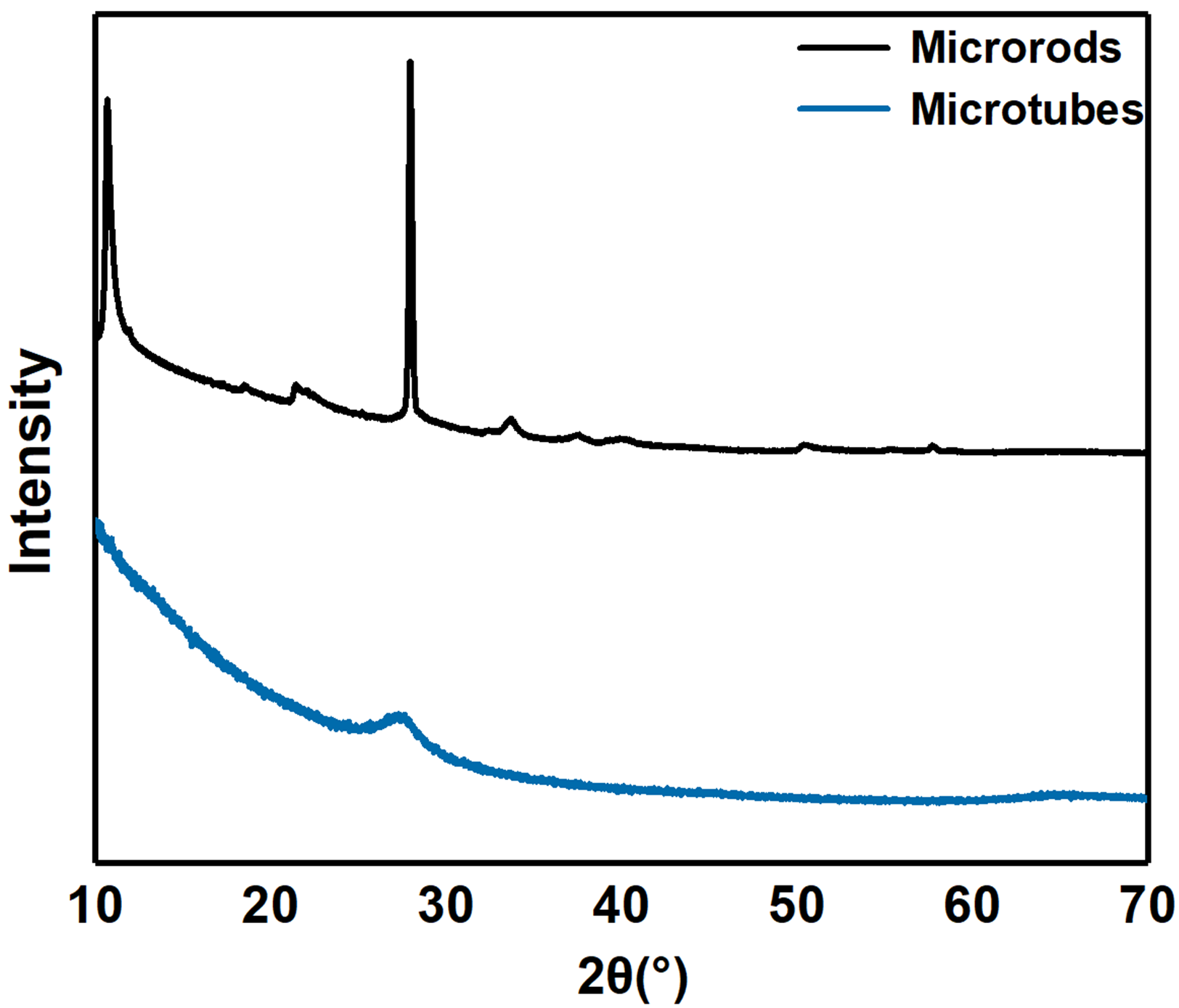

Supplement: Supplementary 1 — Figs. S1 to S9 Videos S1 to S9 [file research.0565.f1.zip › Figure S3.tif]

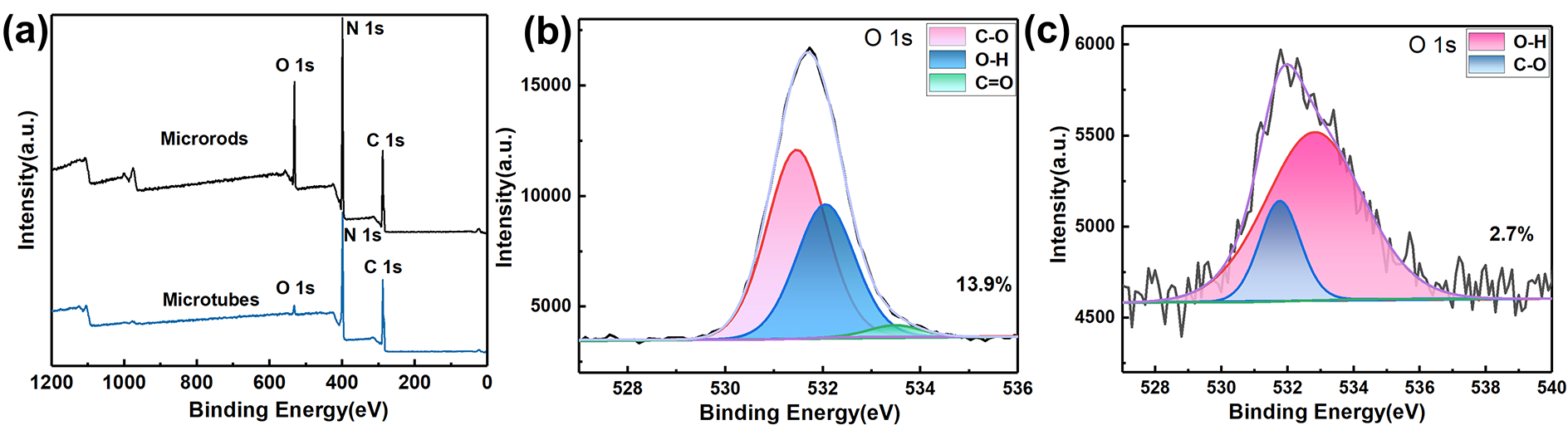

Supplement: Supplementary 1 — Figs. S1 to S9 Videos S1 to S9 [file research.0565.f1.zip › Figure S4.tif]

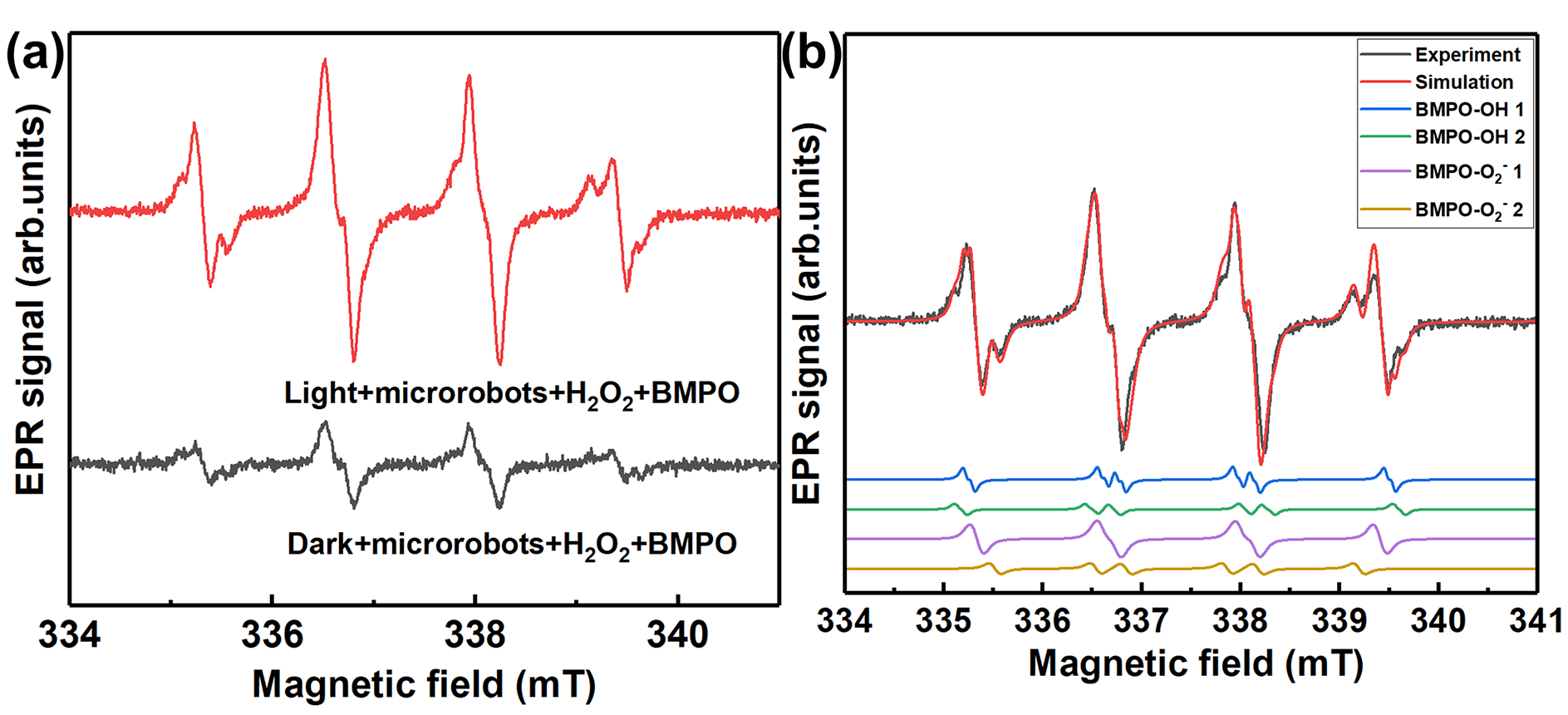

Supplement: Supplementary 1 — Figs. S1 to S9 Videos S1 to S9 [file research.0565.f1.zip › Figure S5.tif]

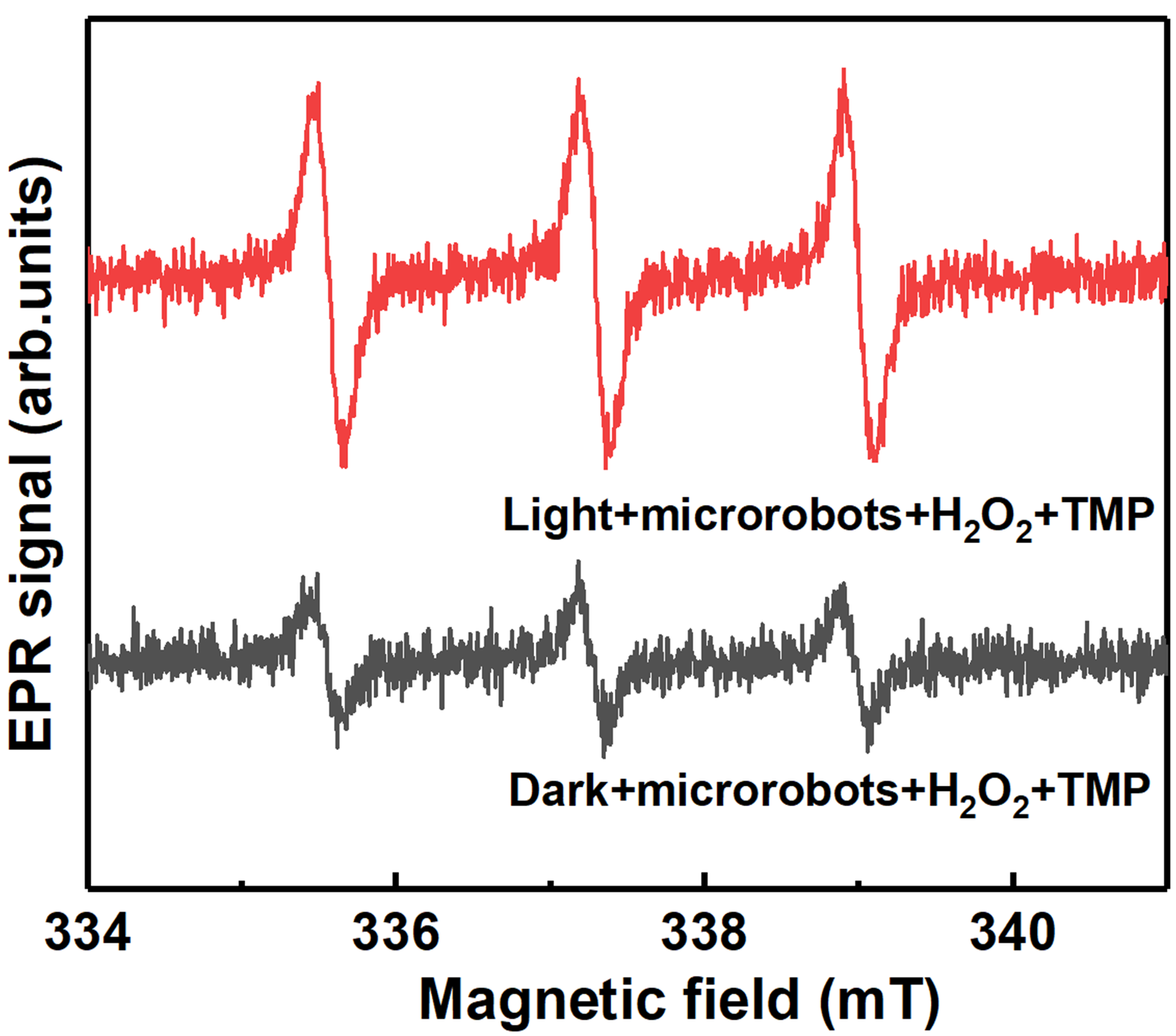

Supplement: Supplementary 1 — Figs. S1 to S9 Videos S1 to S9 [file research.0565.f1.zip › Figure S6.tif]

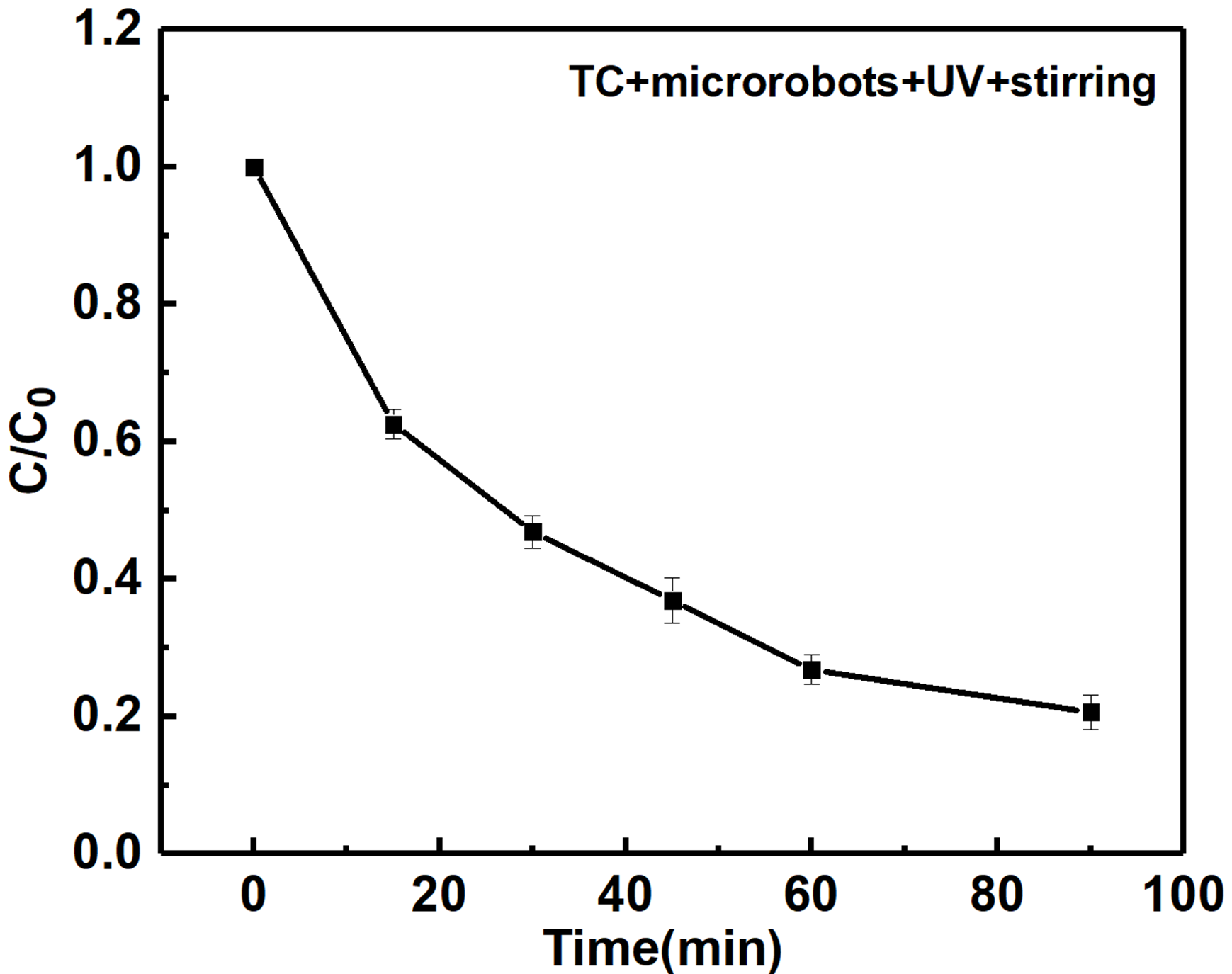

Supplement: Supplementary 1 — Figs. S1 to S9 Videos S1 to S9 [file research.0565.f1.zip › Figure S7.tif]

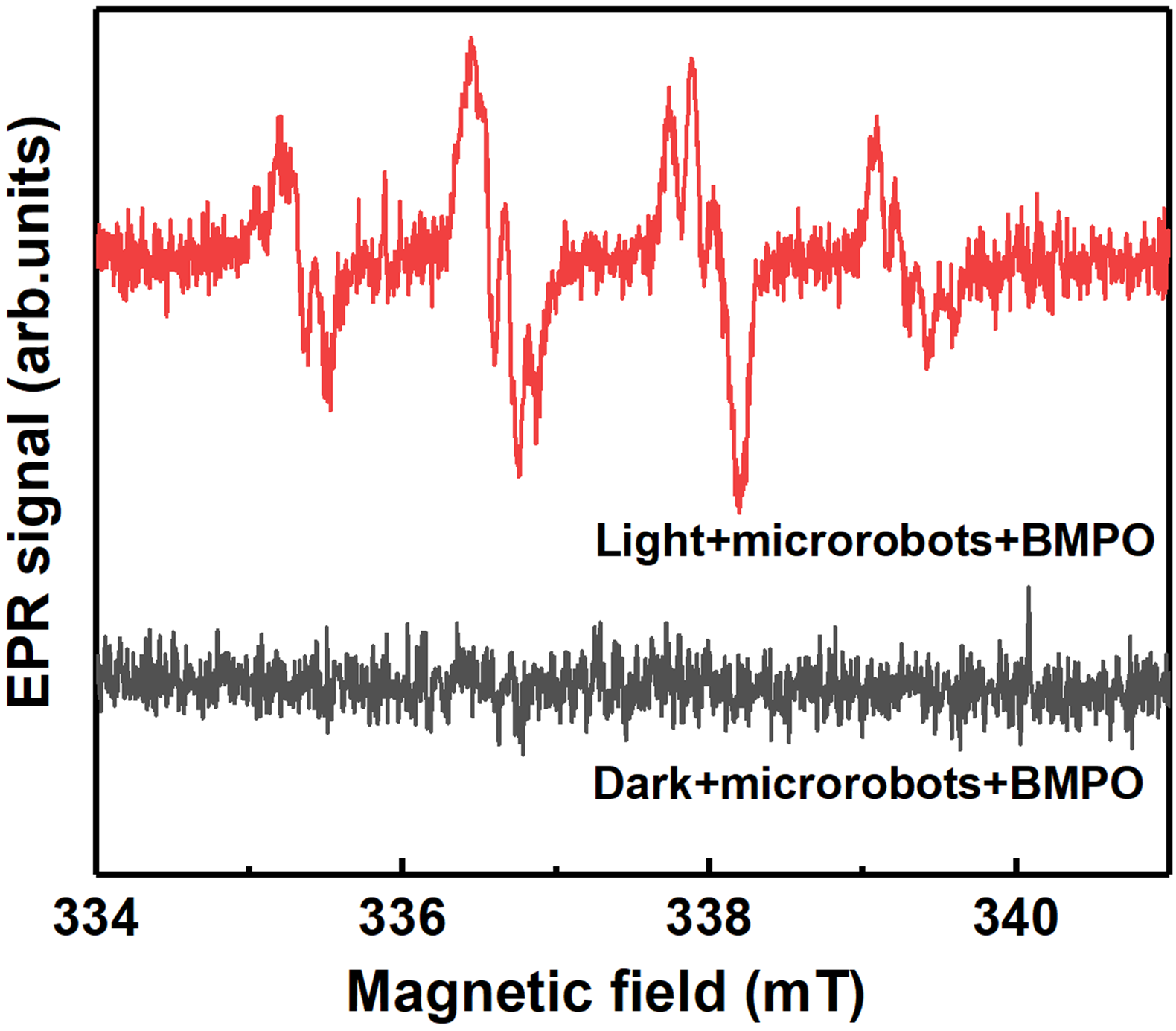

Supplement: Supplementary 1 — Figs. S1 to S9 Videos S1 to S9 [file research.0565.f1.zip › Figure S8.tif]

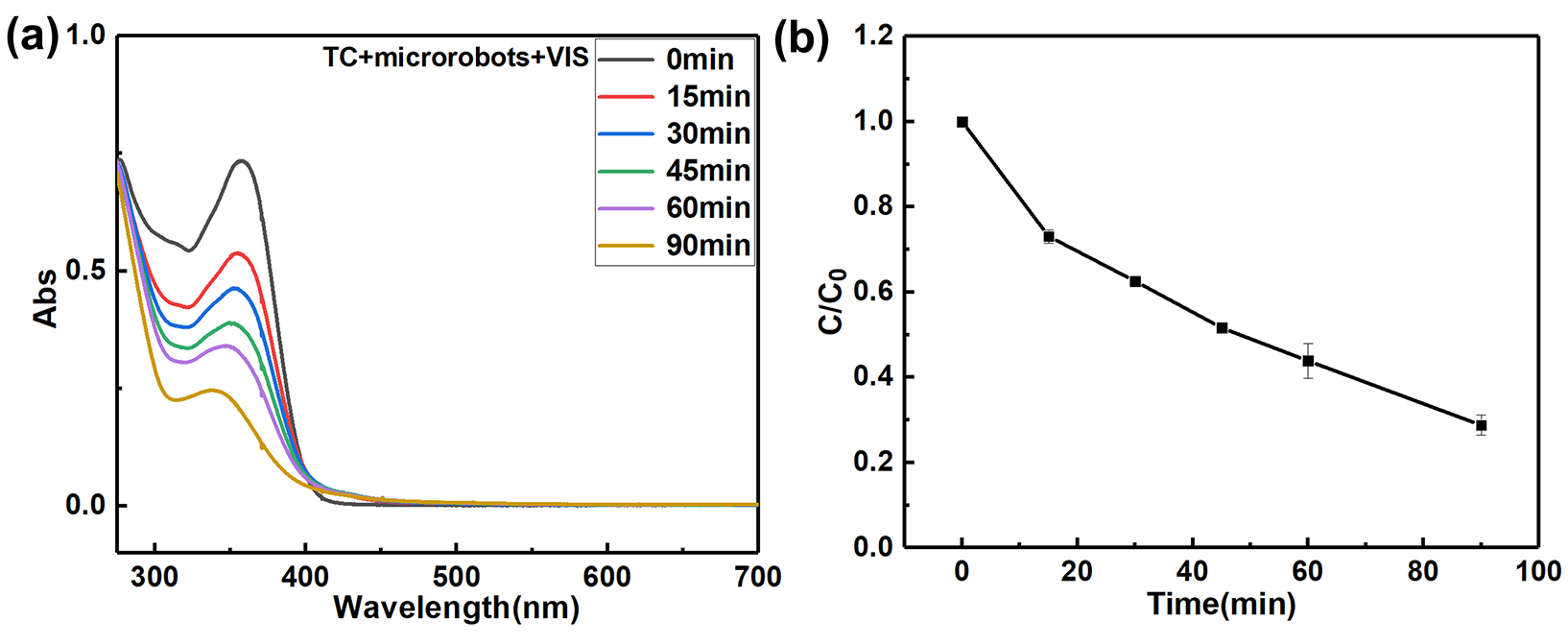

Supplement: Supplementary 1 — Figs. S1 to S9 Videos S1 to S9 [file research.0565.f1.zip › Figure S9.tif]
